# Design, Fabrication and Levitation Experiments of a Micromachined Electrostatically Suspended Six-Axis Accelerometer

**DOI:** 10.3390/s111211206

**Published:** 2011-11-28

**Authors:** Feng Cui, Wu Liu, Wenyuan Chen, Weiping Zhang, Xiaosheng Wu

**Affiliations:** National Key Laboratory of Science and Technology on Nano/Micro Fabrication Technology, Research Institute of Micro/Nano Science and Technology, Shanghai Jiao Tong University, 800 Dongchuan Road, Shanghai 200240, China; E-Mails: sdcuifeng@163.com (F.C.); liuwu@sjtu.edu.cn (W.L.); zwp37@163.com (W.Z.); xswu@sjtu.edu.cn (X.W.)

**Keywords:** MEMS accelerometer, electrostatic suspension, six-axis, hybrid microfabrication, UV-LIGA, SU-8, levitation

## Abstract

A micromachined electrostatically suspended six-axis accelerometer, with a square plate as proof mass housed by a top stator and bottom stator, is presented. The device structure and related techniques concerning its operating principles, such as calculation of capacitances and electrostatic forces/moments, detection and levitation control of the proof mass, acceleration measurement, and structural parameters design, are described. Hybrid MEMS manufacturing techniques, including surface micromachining fabrication of thin film electrodes and interconnections, integration fabrication of thick nickel structures about 500 μm using UV-LIGA by successful removal of SU-8 photoresist mold, DRIE of silicon proof mass in thickness of 450 μm, microassembly and solder bonding, were employed to fabricate this prototype microdevice. A levitation experiment system for the fabricated microaccelerometer chip is introduced, and levitation results show that fast initial levitation within 10 ms and stable full suspension of the proof mass have been successfully demonstrated.

## 1. Introduction

An electrostatically suspended accelerometer mainly comprises a mechanically free proof mass and stator electrodes, which maintain the mass suspension at its null position by capacitive position detection and electrostatic levitation control. The operation principle of this servo-controlled electrostatic accelerometer is based on the measurement of the electrostatic force necessary to maintain the proof-mass motionless with respect to the sensor cage [1]. The electrostatic force gives the possibility to generate very weak but accurate accelerations while the capacitive sensing offers a high position resolution with negligible back-action, which can allow the electrostatic accelerometer to achieve extremely high resolution and high sensitivity. The unique micro-gravity environment in space has brought this high-precision electrostatic accelerometer numerous space applications, such as the measurement of micro-gravity and non-gravitational weak forces induced by atmospheric drag, solar radiation pressure, and so on. Electrostatic space accelerometers, with high resolutions ranging from pico-g to better than femto-g in the low frequency domain below 1 Hz, have been or will be used in many space scientific missions, such as CHAMP, GRACE and GOCE satellite projects for determination of the Earth’s gravity [1], the LISA mission satellites for the observation of the gravity waves [2], and the MICROSCOPE mission for orbit testing of the equivalence principle [3].

Traditional electrostatic space accelerometers, whose proof-mass is usually made of platinum-rhodium alloy or gold coated titanium alloy, are mainly manufactured by accurate machining and grinding, and thus suffer from the problems associated with complicated machining processes, large size and high cost, which limits their potential applications for micro platforms such as micro spacecraft, micro aerial vehicles, unmanned underwater vehicles, small long-range munitions, *etc*. Based on the MEMS technology, an electrostatically suspended micro-accelerometer (ESMA) has the merits of small size, low-power and low cost. Compared to conventional force-balanced micromachined accelerometers, which typically have a proof mass connected to the substrate by a mechanical spring system, the effective spring constant of the ESMA depends only on the levitation voltage of the system due to the electrostatic levitation of the proof mass [4,5]. By changing the bias voltage, the spring constant can be readily adjusted, thus the sensitivity and the bandwidth of the system can be tuned according to different sensor applications. Furthermore, six-axis accelerations, namely three linear accelerations and three angular accelerations, can be measured simultaneously by only one ESMA sensor, where uniform sensitivity in all degrees of freedom can be offered. This six-axis ESMA is very suitable to constitute a micro inertial measurement unit (MIMU), especially a gyroscope-free inertial measurement unit [6].

Although the ESMA has the potential to deliver navigation-grade performance, relatively little work has been done to realize it, due to the difficult techniques required, such as microfabrication, high vacuum packaging, detection and control of six degrees of freedom (6-DoF) of the proof mass. Among these techniques, one of the most challenging is microfabrication, because very small gap spacing, for electrostatic forces or torques generation and capacitive detection, should be formed between the proof mass and the stators. Using proprietary Ball semiconductor technology [7], a single crystal silicon spherical proof mass with 1 mm diameter has been commercially used as an electrostatically levitated 3-axis accelerometer [8], whose noise floor was reported about 40 μg/Hz^1/2^ level. For a flat disc-like proof mass mostly employed, the micromachined electrostatically suspended accelerometer, as well as rotational microgyroscope by rotating the disc proof mass at high speed, have been reported mainly in [4,5,9–12]. Additionally a levitated rotational microgyro/accelerometer prototype with a flat ring-shaped silicon rotor has been successfully developed, whose noise floor of the tri-axis accelerometer was 20 μg/Hz^1/2^ in 10 Hz bandwidth [12]. However, no six-axis ESMA employing MEMS technology with a square micro plate used as the proof mass has been reported so far.

Two main popular micromachining techniques have been investigated to develop the levitated microaccelerometer, that is, high-aspect-ratio electroplating [4,5,9] and bulk microfabrication based on glass/silicon/glass bonding [10–12]. Employing UV-LIGA technology, with patterned SU-8 ultrathick photoresist as plating mold, we have successfully fabricated our electrostatically suspended inertial sensor, where the electroplated nickel rotor released and thick nickel structures integrated in the bottom stator were about in thickness of 200 μm [9]. For fabrication process relied on deep reactive ion etching (DRIE) of silicon and glass/silicon/glass stack bonding, it has demonstrated the potential of the suspended micro-gyroscope/accelerometer [11,12]. However, difficulties occurred during this microfabrication process, such as the difficulty to release the silicon proof mass, which easily sticks to the glass substrate during high voltage anodic bonding, ohmic contact problems between silicon and metal wires, and the RIE lag effect leading to the damage of the proof mass surfaces, making the qualified rate of the sensor device very low [10].

To improve the operational stability and capacitive detection sensitivity of the ESMA in all directions, especially in lateral orientation, thick flat proof mass and high aspect ratio lateral gaps between the proof mass and sidewall electrodes are required. High aspect ratio micromachining methods, like the well-developed bulk silicon DRIE process and non-silicon X-ray LIGA or UV-LIGA process, can be used to fabricate such thick proof mass and sidewall electrodes. Although the X-ray LIGA and DRIE techniques are better processes for high aspect ratio microstructures, advanced equipment as well as complicated fabrication procedures are an evident burden to production cost. The UV-LIGA technique, utilizing ultraviolent (UV) light as exposure energy, can be performed with the standard lithography equipment. As for the ultrathick, high-aspect-ratio MEMS-type applications, the most promising UV-LIGA technology is based on the SU-8 photoresist [13]. It can form very thick resist molds with vertical sidewall profile, and the maximum aspect ratio can be larger than 20 [14].

In this paper, the structural design and detailed operation principles of a six-axis electrostatically suspended microaccelerometer, with a square plate as proof mass housed by a top stator and bottom stator, are presented. Hybrid MEMS manufacturing techniques, including surface micromachining fabrication of thin film electrodes and interconnections, integration fabrication of about 500 μm thick nickel structures using SU-8 UV-LIGA, DRIE of silicon proof mass in thickness of 450 μm, microassembly and solder bonding, were employed to fabricate this sensor prototype. In addition initial levitation and stable suspension of the proof mass for the fabricated ESMA chip were successfully demonstrated. Section 2 of this paper describes the design of the ESMA device and its detailed operating principles. The microfabrication processes of the designed ESMA, including processes of the top stator, bottom stator, silicon proof-mass and reflow bonding, are described in Section 3. Section 4 is the fabrication results and discussion. Levitation experiment system and results for the fabricated ESMA chip are presented in Section 5. Section 6 gives the conclusions of this paper.

## 2. Design and Operation of an ESMA Device

In the following, the designed structure and detailed techniques about the operation principles for the micromachined electrostatically suspended six-axis accelerometer are described.

### 2.1. Designed Structure

To realize stable electrostatic levitation, the electrostatic forces should be generated in such a way that the net potential of the proof mass is held a constant, usually virtual ground, so that by changing potentials of the control electrodes, magnitude and direction of the resultant forces or moments exerting on the proof mass can be precisely adjusted. Since there is no mechanical connection to the substrate, to maintain the proof mass at zero potential and to measure its suspended position, two special approaches are often taken. One approach, employed in the macro electrostatically suspended accelerometer [1], is that a very thin gold wire of 5 μm diameter or less was used to electrically link the proof mass to the cage. Through the gold wire, a biasing voltage and an excitation signal were applied to the proof mass and net charges of the mass can also be discharged, however, stiffness of the gold wire would influence the electrostatic suspension and moreover, its fabrication was challenging and not suitable for micro-scale devices. The other approach is that common electrodes are used to capacitively couple signals to the suspended proof mass, and by applying voltages of the same magnitude and opposite polarity, pair of levitation control electrodes of equal area was utilized to hold net potential of the mass near virtual ground. This approach is employed in most MEMS suspended devices and was also adopted in our ESMA sensor.

**Figure 1.**
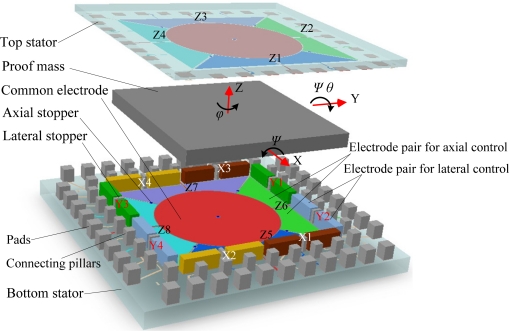
The exploded view of the microaccelerometer configuration.

The structure of an electrostatically suspended six-axis microaccelerometer with a square plate proof mass housed by stator electrodes, assembled using hybrid microfabrication technology, is illustrated in Figure 1.

A square plate, which may have a certain number of through holes (not shown in this figure) to reduce air damping when working at low vacuum environment, is employed as the suspended proof mass. It can be made of silicon fabricated by DRIE or metal employing LIGA-like micromaching methods or conventional ultra-precision machining methods.

A top glass plate and a bottom glass plate are used as substrates for carrying electrodes and interconnections. The stator electrodes, composed of axial levitation electrodes, lateral levitation electrodes and common electrodes, are symmetrically arranged around the proof mass to form capacitors for 6-DoF electrostatic levitation and capacitive detection. Here levitation control electrodes are used for electrostatic forces generation and proof-mass position sensing simultaneously. On the bottom stator and top stator, thin film electrodes, composed of axial levitation electrodes and common electrodes, are formed. Four pairs of axial levitation electrodes at each stator plate, illustrated as Z1∼Z4 at the top stator and Z5∼Z8 at the bottom stator, are symmetrically disposed along the X-axis and Y-axis. These axial levitation electrodes are used to detect and control the proof-mass displacement in three degrees of freedom, which are the translation along the Z-axis and the rotations around the X and Y axes. There is a common electrode located in the center of each plate. Both the common electrodes, used for signal pick-off or exciting, are connected together to form a large common electrode. On the bottom stator, eight pairs of lateral levitation electrodes made of thick electroplating metal, labeled X1∼X4 in the X-axis direction and Y1∼Y4 in the Y-axis direction, are symmetrically distributed around the axial levitation electrodes. The lateral levitation electrodes are detection and control electrodes used for 3-DoF in-plane motion control of the proof mass, that is, the translations along the X and Y axes, and the rotation around the Z-axis.

To prevent large axial and lateral displacements of the proof mass from contacting the stator electrodes, five axial stoppers and eight lateral stoppers, as shown in Figure 1, are mounted on the glass substrates. The stoppers can also be used to overcome initial stiction between the proof mass and the electrodes when the proof mass starts to be levitated. Moreover, the stoppers are conductive so that possible residual charges accumulated in the proof mass can be discharged to the outside when the proof mass is seated.

### 2.2. Operation of the ESMA

To develop a six-axis microaccelerometer, the levitated proof mass must be stably maintained at its null working position relative to the stator. The levitation of the proof mass, in 6-DoF of three translations and three rotations, is servo-controlled by capacitive position detecting and electrostatic actuating through control electrode pairs.

The closed-loop electronic control is used to generate electrostatic forces maintaining the proof mass at the center of the cavity. In the presence of external forces and/or moments, the proof mass displaces away from its nominal position, resulting in a change in the capacitance formed between the levitation control electrodes and the proof mass. The control electronics then detect these capacitance changes and, in turn, apply voltages to the levitation electrodes, generating electrostatic forces in order to rebalance the position of the proof mass. The voltages that are required to generate these balancing forces and/or moments can be used to measure the input accelerations.

To describe the operation of the designed 6-axis ESMA in detail, in the following, the capacitances and electrostatic forces/moments between levitation electrodes and the proof mass, position detection of the proof mass, acceleration measurement and main performance indexes, structural parameters design based on hybrid microfabrication method, are presented.

#### 2.2.1. Capacitances and Electrostatic Forces/Moments Between Levitation Electrodes and the Proof Mass

**Figure 2.**
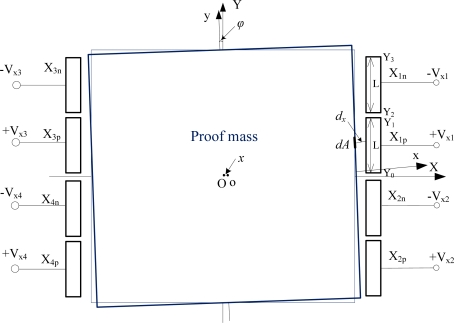
Structural parameters of the accelerometer along X-axis direction.

Here only the lateral levitation electrode pairs X*i*(X_*i*p_, X_*i*n_) (*i* = 1 ∼ 4), distributed along the X axis, as shown in Figure 2, are considered to illustrate calculation of capacitances, electrostatic forces in the X direction, and electrostatic moments around the Z-axis. For the electrode pairs X*i*(X_*i*p_, X_*i*n_) (*i* = 1 ∼ 4), one electrode X_*i*p_ is set to application of positive levitation voltage while the other electrode X_*i*n_ will be applied negative levitation voltage of the same magnitude. The derivative process of capacitances, electrostatic forces/moments for other levitation electrode pairs of Z-axial and Y-axial directions are similar and omitted here.

In Figure 2, the electrode coordinate system is XOY while the xoy coordinate system is fixed at the proof mass, where origins of coordinates O and o are geometric centers of the stator electrode system and the proof mass, respectively. Assuming the proof mass has tiny movements of translation *x* along the X axis and then rotation *ϕ* around the Z-axis, the lateral gap *d_x_* at the small sidewall area *dA* between the sidewalls of the proof mass and the lateral levitation electrode such as X_1p_, is:

(1) $$d_{x}\approx d_{s0}-x+\phi y\;(Y_{0}\leq y\leq Y_{1},\;-d_{s0}<x<d_{s0})$$

where *d_s0_* is the nominal lateral gap when the proof mass is suspended at its null place, *Y_0_* and *Y_1_* are two ends position along Y-axis of the lateral electrode X_1p_.

The capacitance between the lateral levitation electrode X_1p_ and the proof mass is:

(2) $$\begin{array}{ll} C_{x1p} & =ɛ\int_{A}\frac{\mathrm{dA}}{d_{x}}=ɛ\int_{Y_{0}}^{Y_{1}}\;\frac{h}{d_{s0}-x+\phi y}\mathrm{dy}\\ & =ɛh\frac{\text{ln}|d_{s0}-x+\phi Y_{1}|-\;\text{ln}|d_{s0}-x+\phi Y_{0}|}{\phi }\\ & =ɛh\frac{\text{ln}|1-\frac{x-\phi Y_{1}}{d_{s0}}|-\;\text{ln}|1-\frac{x-\phi Y_{0}}{d_{s0}}|}{\phi }\\ & =\frac{ɛh}{\phi }[\frac{\phi (Y_{1}-Y_{0})}{d_{s0}}-{\left(\frac{x-\phi Y_{1}}{d_{s0}}\right)}^{2}/2+{\left(\frac{x-\phi Y_{0}}{d_{s0}}\right)}^{2}/2-{\left(\frac{x-\phi Y_{1}}{d_{s0}}\right)}^{3}/3+{\left(\frac{x-\phi Y_{0}}{d_{s0}}\right)}^{3}/3-...]\\ & \approx ɛh[\frac{Y_{1}-Y_{0}}{d_{s0}}-\frac{\phi (Y_{1}^{2}-Y_{0}^{2})-2x(Y_{1}-Y_{0})}{2d_{s0}{}^{2}}+\frac{3x^{2}(Y_{1}-Y_{0})-3x\phi (Y_{1}^{2}-Y_{0}^{2})+\phi^{2}(Y_{1}^{3}-Y_{0}^{3})}{3d_{s0}{}^{3}}+...] \end{array}$$

where *ɛ* is the air permittivity, *h* is thickness of the proof mass. Since displacements *x* and *ϕ* of the proof mass are small quantities, their second order quantities can be omitted. And considering length of the lateral electrode is *L* = *Y_1_* − *Y_0_*, then:

(3) $$C_{x1p}\approx ɛ\frac{\mathrm{hL}}{d_{s0}}(1+\frac{1}{d_{s0}}x-\frac{Y_{1}+Y_{0}}{2d_{s0}}\phi )$$

In the same way, the capacitance between the lateral levitation electrode X_1n_ and the proof mass is:

(4) $$C_{x1n}=ɛ\int_{Y_{2}}^{Y_{3}}\frac{h}{d_{s0}-x+\phi y}\mathrm{dy}\approx ɛ\frac{\mathrm{hL}}{d_{s0}}[1+\frac{1}{d_{s0}}x-\frac{\left(Y_{3}+Y_{2}\right)}{2d_{s0}}\phi ]$$

where *Y_2_* and *Y_3_* are two ends position along Y-axis of the lateral electrode X_1n_.

Therefore, the linear expression for the total capacitance between the lateral electrode pair X1(X_1p_,X_1n_) and the proof mass is:

(5) $$C_{x1}=C_{x1p}+C_{x1p}\approx C_{s0}(1+{ɛ}_{\mathrm{xN}}-{ɛ}_{\phi N})$$

Here, nominal capacitance between one lateral electrode pair and the proof mass at the null position is:

(6) $$C_{s0}=2ɛ\;\frac{\mathrm{hL}}{d_{s0}}$$

*ɛ_xN_* and *ɛ_ϕN_* are normalized dimensionless displacements:

(7) $${ɛ}_{\mathrm{xN}}=\frac{x}{d_{s0}}{ɛ}_{\phi N}=\frac{Y_{3}+Y_{2}+Y_{1}+Y_{0}}{4d_{s0}}\;\phi$$

In the same way, the linear capacitance expressions between the other three lateral electrode pairs and the proof mass are:

(8) $$\begin{array}{l} C_{x2}=C_{x2p}+C_{x2n}\approx C_{s0}(1+{ɛ}_{\mathrm{xN}}+{ɛ}_{\phi N})\\ C_{x3}=C_{x3p}+C_{x3n}\approx C_{s0}(1-{ɛ}_{\mathrm{xN}}+{ɛ}_{\phi N})\\ C_{x4}=C_{x4p}+C_{x4n}\approx C_{s0}(1-{ɛ}_{\mathrm{xN}}-{ɛ}_{\phi N}) \end{array}$$

So the differential capacitances caused by the translation displacement *x* and rotation displacement *ϕ* of the proof mass, are decoupled, and are expressed as the following:

(9) $$\Delta C_{x}^{X}=C_{x1}+C_{x2}-(C_{x3}+C_{x4})=4C_{s0}{ɛ}_{\mathrm{xN}}$$

(10) $$\Delta C_{x}^{\phi }=C_{x2}+C_{x3}-(C_{x1}+C_{x2})=4C_{s0}\;{ɛ}_{\phi N}$$

Similarly, the linear capacitance expressions between the four lateral electrode pairs Y*i*(Y_*i*p_, Y_*i*n_) (*i* = 1 ∼ 4) and the proof mass are:

(11) $$\begin{array}{l} C_{Y1}=C_{Y2p}+C_{Y2n}\approx C_{s0}(1+{ɛ}_{\mathrm{yN}}-{ɛ}_{\phi N})\\ C_{Y2}=C_{Y2p}+C_{Y2n}\approx C_{s0}(1+{ɛ}_{\mathrm{yN}}+{ɛ}_{\phi N})\\ C_{Y3}=C_{Y3p}+C_{Y3n}\approx C_{s0}(1-{ɛ}_{\mathrm{yN}}+{ɛ}_{\phi N})\\ C_{Y4}=C_{Y4p}+C_{Y4n}\approx C_{s0}(1-{ɛ}_{\mathrm{yN}}-{ɛ}_{\phi N}) \end{array}$$

where *ɛ_yN_* is normalized displacement along Y-axis.

Using the same derivative process, the linear capacitance expressions between the eight axial levitation electrode pairs Z*i*(Z_*i*p_, Z_*i*n_) (*i* = 1 ∼ 8) and the proof mass can be obtained as the following:

(12) $$\begin{array}{ll} C_{Z1}\approx C_{z0}[1+{ɛ}_{\mathrm{zN}}-{ɛ}_{\theta N}] & C_{Z2}\approx C_{z0}[1+{ɛ}_{\mathrm{zN}}+{ɛ}_{\psi N}]\\ C_{Z5}\approx C_{z0}[1-{ɛ}_{\mathrm{zN}}+{ɛ}_{\theta N}] & C_{Z6}\approx C_{z0}[1-{ɛ}_{\mathrm{zN}}-{ɛ}_{\psi N}]\\ C_{Z3}\approx C_{z0}[1+{ɛ}_{\mathrm{zN}}+{ɛ}_{\theta N}] & C_{Z4}\approx C_{z0}[1+{ɛ}_{\mathrm{zN}}-{ɛ}_{\psi N}]\\ C_{Z7}\approx C_{z0}[1-{ɛ}_{\mathrm{zN}}-{ɛ}_{\theta N}] & C_{Z8}\approx C_{z0}[1-{ɛ}_{\mathrm{zN}}+{ɛ}_{\psi N}] \end{array}$$

where *C_Z0_* is nominal capacitance between one axial electrode pair and the proof mass at the null position, *ɛ_zN_*, *ɛ_ψN_* and *ɛ_θN_* are normalized dimensionless displacements along Z-axis, rotation around X-axis and rotation around Y-axis, respectively.

The electrostatic force applied to the proof mass generated by the lateral electrode X_1p_ with actuation voltage of *V_x1_*, is derived from the formula (2) as:

(13) $$F_{x1p}=\frac{1}{2}\;\frac{\partial C_{x1p}}{\partial x}V_{x1}{}^{2}\approx ɛh\frac{L}{2d_{s0}{}^{2}}V_{x1}{}^{2}(1-\frac{Y_{1}+Y_{0}}{d_{s0}}\phi +\frac{2}{d_{s0}}x)$$

As well, the electrostatic force generated by the electrode X_1n_ is:

(14) $$F_{x1n}=\frac{1}{2}\;\frac{\partial C_{x1n}}{\partial x}V_{x1}{}^{2}\approx ɛh\frac{L}{2d_{s0}{}^{2}}V_{x1}{}^{2}(1-\frac{Y_{2}+Y_{3}}{d_{s0}}\phi +\frac{2}{d_{s0}}x)$$

Therefore the electrostatic force generated by the lateral electrode pair (X_1p_, X_1n_) is:

(15) $$\begin{array}{ll} F_{x1} & =F_{x1p}+F_{x1n}\\ & \approx ɛh\frac{L}{d_{s0}{}^{2}}V_{s0}{}^{2}(1-\frac{Y_{0}+Y_{1}+Y_{2}+Y_{3}}{2d_{s0}}\phi +\frac{2}{d_{s0}}x)\frac{V_{x1}{}^{2}}{V_{s0}{}^{2}}\\ & =F_{x0}(1+2{ɛ}_{\mathrm{xN}}-2{ɛ}_{\phi N})u_{x1}{}^{2} \end{array}$$

Here *V_s0_* is bias voltage for lateral levitation control electrodes, nominal electrostatic force, generated by the lateral electrode pair when the proof mass is suspended at the null place, is:

(16) $$F_{x0}=ɛh\frac{L}{d_{s0}{}^{2}}V_{s0}{}^{2}$$

and the normalized dimensionless voltages applied on the levitation electrodes are defined as:

(17) $$u_{\mathrm{xi}}{}^{2}=\frac{V_{\mathrm{xi}}{}^{2}}{V_{s0}{}^{2}}\;\;\;(i=1,2,3,4)$$

In the same way, the electrostatic forces generated by the other three lateral electrode pairs are:

(18) $$\begin{array}{l} F_{x2}\approx F_{x0}(1+2{ɛ}_{\mathrm{xN}}+2{ɛ}_{\phi N})u_{x2}{}^{2}\\ F_{x3}\approx -F_{x0}(1-2{ɛ}_{\mathrm{xN}}+2{ɛ}_{\phi N})u_{x3}{}^{2}\\ F_{x4}\approx -F_{x0}(1-2{ɛ}_{\mathrm{xN}}-2{ɛ}_{\phi N})u_{x4}{}^{2} \end{array}$$

For the voltage applied to the levitation electrode, a preload bias voltage superimposed feedback voltage, which is generated by a controller according to displacements of the proof mass, is employed. Assuming the feedback voltage that makes the proof mass have positive linear or angular displacement, in right-hand rule, is positive, the voltages applied to the lateral levitation electrode pairs X*i*(X_*i*p_, X_*i*n_) (*i* = 1,2,3,4) are expressed as the following:

(19) $$\begin{array}{l} V_{x1}=V_{s0}+U_{x}-U_{\phi }\\ V_{x2}=V_{s0}+U_{x}+U_{\phi }\\ V_{x3}=V_{s0}-U_{x}+U_{\phi }\\ V_{x4}=V_{s0}-U_{x}-U_{\phi } \end{array}$$

where *U_x_* and *U_ϕ_* are feedback voltages induced by displacements *x* and *ϕ* of the proof mass, respectively. According to expression (17), the above expression is normalized as:

(20) $$\begin{array}{l} u_{x1}=1+u_{x}-u_{\phi }\\ u_{x2}=1+u_{x}+u_{\phi }\\ u_{x3}=1-u_{x}+u_{\phi }\\ u_{x4}=1-u_{x}-u_{\phi } \end{array}$$

So, the resultant electrostatic force along the X axis generated by the lateral levitation electrode pairs (X_*i*p_, X_*i*n_) (*i* = 1,2,3,4) is:

(21) $$F^{X}=\sum_{i=1}^{4}F_{\mathrm{xi}}=8F_{x0}[u_{x}+{ɛ}_{\mathrm{xN}}(1+u_{x}^{2}+u_{\phi }^{2})+2{ɛ}_{\phi N}u_{x}u_{\phi }]$$

**Figure 3.**
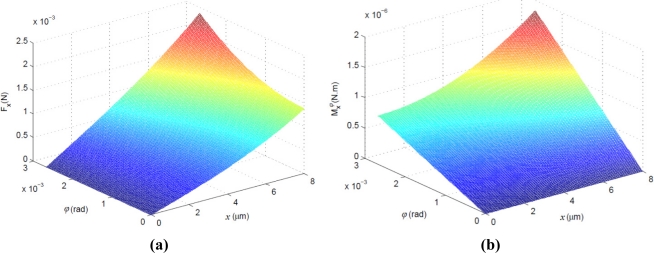
Surface plots of **(a)** electrostatic force *F^X^*(*x*, *ϕ*) of formula (21) and **(b)** electrostatic moment $M_{x}{}^{\phi }(x,\;\phi )$ of formula (30) for an ESMA with structural parameters shown in Table 1 (here *V_s0_* = 40 V, *d_s0_* = 10 μm).

This is a one order model about electrostatic force and displacements of the proof mass, where axis-coupling between *x* and *ϕ* is considered, however, it can describe movement of the proof mass more accurately. In Figure 3(a), a surface plot of electrostatic force *F^X^*(*x*, *ϕ*) of formula (21), for an ESMA with a proof mass of 6 mm × 6 mm × 450 μm and lateral gap of 10 μm, shows that when the displacements *x* and *ϕ* of the proof mass are large near the maximum range of its motion, the degree of nonlinearity of the *F^X^*(*x*, *ϕ*) is serious, and when the *x* and *ϕ* are small, the influence of coupling-axis angular displacement *ϕ* on the electrostatic force *F^X^* is negligible.

When the active electrostatic bearing system of the micro-accelerometer is working at the null place, the *ɛ_xN_*, *ɛ_ϕN_*, *u_ϕ_* and *u_ϕ_* of the proof mass around its nominal point are very small, therefore:

(22) $$F^{X}\approx 8F_{x0}(u_{x}+{ɛ}_{\mathrm{xN}})=\frac{8ɛ\mathrm{hL}}{d_{s0}{}^{2}}(V_{s0}U_{x}+\frac{V_{s0}{}^{2}}{d_{s0}}x)$$

This is a linear and decoupled model of electrostatic force and displacements of the proof mass, which is often used as control model of the electrostatic bearing system and by combining PID controller using different control algorithms, the performance parameters of the system such as frequency characteristics, bandwidth et al could be designed [15–17]. If the translation *x* of the proof mass at the balanced working position is small enough, above electrostatic force is reduced to:

(23) $$F^{X}\approx \frac{8ɛ\mathrm{hL}}{d_{s0}{}^{2}}V_{s0}U_{x}$$

According to the formula (2), the electrostatic moment applied to the proof mass, generated by the lateral electrode X_1p_ with actuation voltage of *V_x1_*, is:

(24) $$M_{x1p}=\frac{1}{2}\frac{\partial C_{x1p}}{\partial \phi }V_{x1}{}^{2}\approx \frac{1}{2}V_{x1}{}^{2}ɛ\mathrm{hL}\frac{Y_{1}+Y_{0}}{2d_{s0}{}^{2}}[-1-\frac{2}{d_{s0}}x+\frac{4(Y_{1}^{3}-Y_{0}^{3})}{3d_{s0}L}\phi ]$$

As well, the electrostatic moment generated by the electrode X_1n_ is:

(25) $$M_{x1n}=\frac{1}{2}\frac{\partial C_{x1n}}{\partial \phi }V_{x1}{}^{2}\approx \frac{1}{2}V_{x1}{}^{2}ɛ\mathrm{hL}\frac{Y_{3}+Y_{2}}{2d_{s0}{}^{2}}[-1-\frac{2}{d_{s0}}x+\frac{4(Y_{3}^{3}-Y_{2}^{3})}{3d_{s0}L}\phi ]$$

Therefore the electrostatic moment generated by the lateral electrode pair X1 (X_1p_, X_1n_) is:

(26) $$\begin{array}{ll} M_{x1} & =M_{x1p}+M_{x1n}\approx \frac{1}{4}ɛ\mathrm{hL}\frac{V_{s0}{}^{2}}{d_{s0}{}^{2}}\frac{V_{x1}{}^{2}}{V_{s0}{}^{2}}[(-1-\frac{2}{d_{s0}}x)(Y_{1}+Y_{0}+Y_{3}+Y_{2})+\frac{4(Y_{1}^{3}-Y_{0}^{3}+Y_{3}^{3}-Y_{2}^{3})}{3d_{s0}L}\phi ]\\ & =\frac{1}{4}F_{x0}(Y_{1}+Y_{0}+Y_{3}+Y_{2})[-1-\frac{2}{d_{s0}}x+\frac{4(Y_{1}^{3}-Y_{0}^{3}+Y_{3}^{3}-Y_{2}^{3})}{3d_{s0}L(Y_{1}+Y_{0}+Y_{3}+Y_{2})}\phi ]\frac{V_{x1}{}^{2}}{V_{s0}{}^{2}}\\ & =M_{x0}[-1-2{ɛ}_{\mathrm{xN}}+\kappa_{\phi N}]u_{x1}{}^{2} \end{array}$$

Here:

(27) $$M_{x0}=\frac{1}{4}F_{x0}(Y_{1}+Y_{0}+Y_{3}+Y_{2})$$

(28) $$\kappa_{\phi N}=\frac{4(Y_{1}^{3}-Y_{0}^{3}+Y_{3}^{3}-Y_{2}^{3})}{3d_{s0}L(Y_{1}+Y_{0}+Y_{3}+Y_{2})}\phi$$

In the same way, the electrostatic moments generated by the other three lateral electrode pairs are:

(29) $$\begin{array}{l} M_{x2}=M_{x2p}+M_{x2n}\approx M_{x0}[1+2{ɛ}_{\mathrm{xN}}+\kappa_{\phi N}]u_{x2}{}^{2}\\ M_{x3}=M_{x3p}+M_{x3n}\approx M_{x0}[1-2{ɛ}_{\mathrm{xN}}+\kappa_{\phi N}]u_{x3}{}^{2}\\ M_{x4}=M_{x4p}+M_{x4n}\approx M_{x0}[-1+2{ɛ}_{\mathrm{xN}}+\kappa_{\phi N}]u_{x4}{}^{2} \end{array}$$

The resultant electrostatic moment around the Z axis generated by the lateral levitation electrode pairs X*i*(X_*i*p_, X_*i*n_) (*i* = 1 ∼ 4) to the proof mass is:

(30) $$M_{x}{}^{\phi }=\sum_{i=1}^{4}M_{\mathrm{xi}}\approx M_{x0}[8u_{\phi }+16{ɛ}_{\mathrm{xN}}u_{x}u_{\phi }+4\kappa_{\phi N}(1+u_{x}{}^{2}+u_{\phi }{}^{2})]$$

This is a one order model with respect to the electrostatic moment and axis-coupling displacements of the proof mass, which is used to accurately describe angular movement of the proof mass. Like the electrostatic force *F^X^*(*x*, *ϕ*), Figure 3(b) shows that when the displacements *x* and *ϕ* of the proof mass are large*,* the degree of nonlinearity of the electrostatic moment $M_{x}{}^{\phi }(x,\;\phi )$ is serious, and when the *x* and *ϕ* are small, the influence of coupling-axis displacement *x* on the electrostatic moment $M_{x}{}^{\phi }$ is negligible.

When the micro-accelerometer is working at the null place, the small second order quantities of the *ɛ_xN_*, *ɛ_ϕN_*, *u_ϕ_* and *u_ϕ_* are negligible, therefore, we get a linear and decoupled model about electrostatic moment and angular displacement of the proof mass:

(31) $$M_{x}{}^{\phi }\approx M_{x0}(8u_{\phi }+4\kappa_{\phi N})=4M_{x0}[\frac{2U_{\phi }}{V_{s0}}+\frac{4(Y_{1}^{3}-Y_{0}^{3}+Y_{3}^{3}-Y_{2}^{3})}{3d_{s0}L(Y_{1}+Y_{0}+Y_{3}+Y_{2})}\phi ]$$

which can be used as angular position control model of the electrostatic bearing system. If the angular displacement *ϕ* of the proof mass at the balanced working position is small enough, above electrostatic moment is reduced to:

(32) $$M_{x}{}^{\phi }\approx 8M_{x0}\frac{U_{\phi }}{V_{s0}}=2ɛh\frac{L}{d_{s0}{}^{2}}(Y_{1}+Y_{0}+Y_{3}+Y_{2})V_{s0}\;U_{\phi }$$

According to geometry symmetry and the same derivative process as the X-axial lateral electrode pairs, we can get similar linear and decoupled model as (31) about electrostatic moment $M_{y}^{\phi }$, generated by the Y-axial lateral electrode pairs, here only its reduced model is written as:

(33) $$M_{y}{}^{\phi }\approx 8M_{y0}\frac{U_{\phi }}{V_{s0}}=8M_{x0}\frac{U_{\phi }}{V_{s0}}=2ɛh\frac{L}{d_{s0}{}^{2}}(Y_{1}+Y_{0}+Y_{3}+Y_{2})V_{s0}\;U_{\phi }$$

Therefore the total electrostatic torque *M^ϕ^*, generated by the X-axial and Y-axial distributed lateral electrode pairs, is:

(34) $$M^{\phi }=M_{x}{}^{\phi }+M_{y}{}^{\phi }\approx 4ɛh\frac{L}{d_{s0}{}^{2}}(Y_{1}+Y_{0}+Y_{3}+Y_{2})V_{s0}\;U_{\phi }$$

The electrostatic forces along Y-axis and Z-axis, and total electrostatic moments around X-axis and Y-axis can be derived in the same way.

#### 2.2.2. Position Detection of the Proof Mass

To detect displacement signals of the ESMA, two capacitive detecting schemes can be applied to the same accelerometer structure. One detecting scheme is the frequency multiplex method with common electrodes used for signal pickoff [11,12], where, by applying high frequency sensing signals to the control electrode pairs, the differential capacitance detecting signals, which indicate the position of the proof mass along the multiple axes, are obtained from the common electrode via the proof mass. On the contrary, the other detection scheme is that the common electrodes are used for excitation by single frequency carrier [10], where the excitation voltage is capacitively coupled to the proof mass through the common electrode. When the proof mass displaces away from its nominal position, differential capacitance between the two corresponding control electrode pairs and the proof mass is detected.

**Figure 4.**
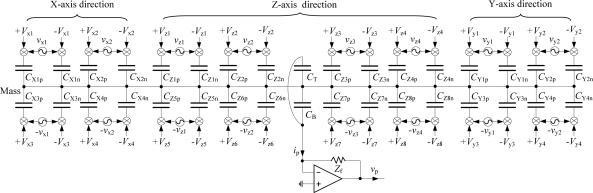
Equvalent capacitive circuits of the micro-accelerometer when common electrodes are used for pickoff.

In this paper, the frequency multiplex detecting method is employed. For the electrodes structure of the microaccelerometer shown in Figure 1, equivalent capacitive detection circuits of the 6-DoF microaccelerometer, for the detecting scheme with common electrodes used for signal pickoff, are shown in Figure 4. Besides DC actuation voltages of the same magnitude and opposite polarity applied to the control electrode pair, a hybrid frequency sensing AC signal, with different high frequency representing linear and angular displacements of the proof mass, is superimposed to the same control electrode pair. Take a lateral electrode pair X1(X_1p_, X_1n_) for example, the actuation voltages +*V_x1_* and –*V_x1_* are applied to the electrodes X_1p_ and X_1n_ respectively, meanwhile a sensing voltage *v_x1_*, which includes carriers of different detecting frequency for translation *x* and for rotation *ϕ* around Z-axis, is superimposed to the *+V_x1_* and −*V_x1_* respectively. Moreover, the two pairs of control electrode, that comprise differential capacitance, have sensing signals of the same magnitude but phase-inverted. In this way, under excitation of carrier signals with different high frequency representing 6-DoF movements of the proof mass, when it deflects from its null balanced position, the differential capacitance detecting signals will be obtained from the common electrode via the proof mass.

The top and bottom common electrodes are electrically connected together, so a total common capacitance between a common pick-up electrode and the proof mass is *C_c_* = *C_T_* + *C_B_*. The displacement detection alternating-current (AC) *i_p_* drawn from the common pick-up electrode, which is then supplied to the virtually grounded input port of a preamplifier, is expressed as:

(35) $$i_{p}=j\omega (C_{T}+C_{B})(v_{m}-0)$$

where *v_m_* is the proof mass potential. When the proof mass has deflections from its null position, the resultant proof mass potential *v_m_* is mainly caused by AC exciting signals applied to the control electrode pairs, which is expressed as:

(36) $$v_{m}=\frac{\sum_{k=1}^{8}v_{\mathrm{zk}}C_{\mathrm{zk}}+\sum_{i=1}^{4}(v_{\mathrm{xi}}\;C_{\mathrm{xi}}+v_{\mathrm{yi}}\;C_{\mathrm{yi}})}{\sum_{k=1}^{8}C_{\mathrm{zk}}+\sum_{i=1}^{4}(C_{\mathrm{xi}}+C_{\mathrm{yi}})+C_{T}+C_{B}}$$

Set Laplacian operator *s* = *jω*, so the detection current (35) is written as:

(37) $$\left\{ \begin{array}{l} i_{p}=K_{c}[\sum_{k=1}^{8}{\mathrm{sv}}_{\mathrm{zk}}\;C_{\mathrm{zk}}+\sum_{i=1}^{4}s(v_{\mathrm{xi}}\;C_{\mathrm{xi}}+v_{\mathrm{yi}}\;C_{\mathrm{yi}})]\\ K_{c}=\frac{C_{T}+C_{B}}{\sum_{k=1}^{8}C_{\mathrm{zk}}+\sum_{i=1}^{4}(C_{\mathrm{xi}}+C_{\mathrm{yi}})+C_{T}+C_{B}} \end{array}\right.$$

where *K_c_* is a proportional constant less than one.

Assuming (*v_x_*, *v_y_*, *v_z_*, *v_ψ_*, *v_θ_*, *v_ϕ_)* are carrier excitation voltages corresponding to 6-DoF displacements (*x,y,z,ψ,θ,ϕ*) of the proof mass, they can be expressed as:

(38) $$\left\{ \begin{array}{l} -v_{j}=V_{s}\;\text{cos}(\omega_{j}t+\eta_{j})\\ +v_{j}=V_{s}\;\text{cos}(\omega_{j}t+\zeta_{j})\\ \eta_{j}=\zeta_{j}\pm 180{}^{\circ} \end{array}\right.(j=x,y,z,\psi ,\theta ,\phi )$$

Here they have six different frequencies, and the carrier signals applied to differential electrode pairs are of the same magnitude but phase-inverted. Then, the hybrid-frequency sensing signal applied to each control electrode pair is expressed as:

(39) $$\begin{array}{llll} v_{z1}=-v_{\theta }+v_{z} & v_{z2}=+v_{\psi }+v_{z} & v_{x1}=+v_{x}-v_{\phi } & v_{y1}=+v_{y}-v_{\phi }\\ v_{z5}=+v_{\theta }-v_{z} & v_{z6}=-v_{\psi }-v_{z} & v_{x2}=+v_{x}+v_{\phi } & v_{y2}=+v_{y}+v_{\phi }\\ v_{z3}=+v_{\theta }+v_{z} & v_{z4}=-v_{\psi }+v_{z} & v_{x3}=-v_{x}+v_{\phi } & v_{y3}=-v_{y}+v_{\phi }\\ v_{z7}=-v_{\theta }-v_{z} & v_{z8}=+v_{\psi }-v_{z} & v_{x4}=-v_{x}-v_{\phi } & v_{y4}=-v_{y}-v_{\phi } \end{array}$$

When the proof mass is suspended at its null position by electrostatic bearing system, its 6-DoF displacements (*x,y,z,ψ,θ,ϕ*) are small quantities, so the linear capacitance expressions (5), (8), (11) and (12) are employed and inserted in the formula (37), then the detection current *i_p_* drawn from the common electrode is:

(40) $$\left\{ \begin{array}{l} i_{p}=4K_{c}s(v_{x}\;C_{s0}\;{ɛ}_{\mathrm{xN}}+v_{y}\;C_{s0}\;{ɛ}_{\mathrm{yN}}+2v_{z}\;C_{z0}\;{ɛ}_{\mathrm{zN}}+v_{\psi }\;C_{z0}\;{ɛ}_{\psi N}+v_{\theta }\;C_{z0}\;{ɛ}_{\theta N}+2v_{\phi }\;C_{s0}\;{ɛ}_{\phi N})\\ K_{c}=\frac{C_{C0}}{8(C_{z0}+C_{s0})+C_{C0}} \end{array}\right.$$

where C_c0_ are the nominal capacitances of common electrodes, for small displacements of the proof mass, *C_T_* + *C_B_* ≈ *C_c0_*. It can be seen that the main way to improve the detection current *i_p_* is to increase *K_c_C_z0_* and *K_c_C_r0_*, so the common electrodes and levitation control electrodes can be optimized for maximum pick-off signal.

The above displacement-detection *i_p_* is supplied to the preamplifier through a resistor with impedance *Z_f_*, and converted into a displacement-detection AC voltage *v_p_*. Such *v_p_* is represented by the following equation:

(41) $$v_{p}=-Z_{f}\;i_{p}=\sum_{j=x,y,z,\psi ,\theta ,\phi }K_{c}Z_{f}\;v_{j}{ɛ}_{\mathrm{jN}}=\sum_{j=x,y,z,\psi ,\theta ,\phi }K_{c}Z_{f}\;V_{s}\omega_{j}{ɛ}_{\mathrm{jN}}\;\text{sin}(\omega_{j}t+\varsigma_{j})$$

It can be seen that the output voltage *v_p_* independently includes all the displacements of the proof mass. Futhermore, the voltage *v_p_* is amplitude-modulated according to individual displacement-detection frequencies *ω_j_*(*j* = *x,y,z,ψ,θ,ϕ*) corresponding to 6-DoF displacements of the proof mass. That is, even when two or more of linear or angular displacements are superimposed, each displacement can be obtained by synchronous demodulation to deliver a controlling signal for the corresponding axis.

#### 2.2.3. Acceleration Measurement and Main Performance Indexes of the Micro-Accelerometer

According to the force balance condition:

(42) F=ma

where m is the mass of the proof mass, **a** is the applied linear acceleration, we take the input acceleration *a_x_* along X-axis direction for example, by substituting resultant X-axial electrostatic force expression (23) into (42), the relationship between feedback voltage *U_x_* and *a_x_* can be obtained:

(43) $$a_{x}\approx \frac{8ɛ\mathrm{hL}}{{\mathrm{md}}_{s0}{}^{2}}V_{s0}U_{x}=\frac{8ɛ\mathrm{hL}}{\rho {\mathrm{hl}}^{2}d_{s0}{}^{2}}V_{s0}U_{x}=\frac{8ɛL}{\rho l^{2}d_{s0}{}^{2}}V_{s0}U_{x}$$

where *l* and *h* are side length and thickness of the square proof-mass block respectively, and *ρ* is its density. In the same way, we can get similar relationships between the feedback voltage *U_z_* and Z-axial acceleration *a_z_*, *U_y_* and Y-axial acceleration *a_y_*, which are omitted here.

When the proof mass of the accelerometer is actively suspended at its working position, the moment balance condition for measuring applied angular acceleration **a** is:

(44) M=Ja

where *J* is moment of inertia of the proof mass. For dimensions *l* × *l* × *h* of the proof mass, the moment of inertia around its z-axis is *J_z_* = *ρl^4^h/6.* According to the resultant electrostatic moment expression around Z-axis (34), we can get the relationship between feedback voltage *U_ϕ_* and the inputted angular acceleration *a_ϕ_* around the Z-axis:

(45) $$a_{\phi }=\frac{M^{\phi }}{J_{z}}\approx \frac{4ɛ\mathrm{hL}(Y_{0}+Y_{2}+Y_{1}+Y_{3})}{J_{z}d_{s0}{}^{2}}V_{s0}U_{\phi }=\frac{24ɛ\mathrm{Lh}(Y_{0}+Y_{2}+Y_{1}+Y_{3})}{{\mathrm{ml}}^{2}d_{s0}{}^{2}}V_{s0}U_{\phi }$$

Similar relationships between the feedback voltage *U_ψ_* and angular acceleration *a_ψ_* around X-axis, *U_θ_* and angular acceleration *a_θ_* around Y-axis, can be achieved.

From expressions (43) and (45), we can see that the inputted accelerations *a_x_* and *a_ϕ_* are linearly related to the feedback voltages *U_x_* and *U_ϕ_*, respectively. Thus, the accelerations applied on the proof mass can be detected through the measurement of the feedback voltages.

When the feedback voltage is equal to the bias voltage, the proof mass is subjected to the maximum of electrostatic force/moment. Thus, the maximum measurable acceleration of the accelerometer can be theoretically calculated as:

(46) $$\begin{array}{l} a_{x\_\text{max}}=\frac{F^{X}{}_{\text{max}}}{m}\approx \frac{8ɛ\mathrm{hL}}{{\mathrm{md}}_{s0}{}^{2}}V_{s0}{}^{2}=\frac{8ɛL}{\rho l^{2}d_{s0}{}^{2}}V_{s0}{}^{2}\\ a_{\phi \_\text{max}}=\frac{M^{\phi }{}_{\text{max}}}{J_{z}}\approx \frac{24ɛ\mathrm{Lh}(Y_{0}+Y_{2}+Y_{1}+Y_{3})}{{\mathrm{ml}}^{2}d_{s0}{}^{2}}V_{s0}{}^{2}=\frac{24ɛL(Y_{0}+Y_{2}+Y_{1}+Y_{3})}{\rho l^{4}d_{s0}{}^{2}}V_{s0}{}^{2} \end{array}$$

Thus, by increasing preload bias voltage, the maximum measurable acceleration can be improved, however, the upper bound of allowable actuation voltage is set by electric breakdown voltage between the proof mass and the associated electrodes. According to the modified Paschen curve for MEMS structures [18], at atmospheric pressure, the electric breakdown voltage is more than 350 V for gap spacing of 4∼10 μm, which is sufficient to achieve large measurable acceleration for most applications. On the other hand when the structural dimensions of the accelerometer are determined, maximum operation range can be improved by employing the proof mass made of lower density material. The detection sensitivity of the sensor, for instance of measuring *a_x_* and *a_ϕ_* accelerations, is:

(47) $$\begin{array}{l} k_{x}=\frac{U_{x}}{a_{x}}\approx \frac{{\mathrm{md}}_{s0}{}^{2}}{8ɛ\mathrm{hL}V_{s0}}=\frac{\rho l^{2}d_{s0}{}^{2}}{8ɛ{\mathrm{LV}}_{s0}}\\ k_{\phi }=\frac{U_{\phi }}{a_{\phi }}\approx \frac{{\mathrm{ml}}^{2}d_{s0}{}^{2}}{24ɛ\mathrm{Lh}(Y_{0}+Y_{2}+Y_{1}+Y_{3})V_{s0}}=\frac{\rho l^{4}d_{s0}{}^{2}}{24ɛL(Y_{0}+Y_{2}+Y_{1}+Y_{3})V_{s0}} \end{array}$$

It can be seen that the sensitivity *k_x_* or *k_ϕ_* is proportional to mass of the proof mass and square of the nominal capacitance gap, but inversely proportional to the preload bias voltage. When the structural parameters of the sensor are determined, the detection sensitivity can be adjusted by changing the applied bias voltage. The resolution of the accelerometer is the smallest acceleration change it can detect, which can be expressed as:

(48) $$\begin{array}{l} a_{x\_\text{min}=}\frac{U_{x\_\text{min}}}{k_{x}}\approx \frac{8ɛ\mathrm{hL}}{{\mathrm{md}}_{s0}{}^{2}}V_{s0}\;U_{x\_\text{min}}\\ a_{\phi \_\text{min}}=\frac{U_{\phi \_\text{min}}}{k_{\phi }}\approx \frac{24ɛ\mathrm{Lh}(Y_{0}+Y_{2}+Y_{1}+Y_{3})V_{s0}}{{\mathrm{ml}}^{2}d_{s0}{}^{2}}U_{\phi \_\text{min}} \end{array}$$

By designing large sensing capacitive gaps and employing a large mass *m*, we can improve the resolution of the microaccelerometer. However, for the structural parameters of the sensor given, the detection resolution is mainly determined by the minimum feedback voltage, which can be expressed as the product of the minimum detectable capacitance variance and gain of the capacitive detection circuits. The minimum detectable capacitance variance is mainly related to the mechanical noise of the sensor, electrical noise and bandwidth of detection circuits, which can reach sub $\text{aF}/\sqrt{\mathrm{Hz}}$ [19,20]. The electronic noise includes random fluctuations in electrical signals for all electronic circuits, which consist mostly of thermal noise, shot noise, and flicker noise. The mechanical noise is mainly thermomechanical noise caused by Brownian motion, which is due to the dynamic unbalanced forces caused by random impacts of molecules on small MEMS structure [21]. When the sensor is working under vacuum conditions, Brownian motion of the gas molecules surrounding the large proof mass can be very low, and the resolution of the sensor is usually limited by the readout electronic noise [22]. In such vacuum environment, due to lack of air damping, electric damping provided by PID controller is usually needed to improve dynamic response performance of levitation system [15–17].

Remarkably, the detection sensitivity varies inversely with the maximum measurable acceleration, that is, when operation range of the accelerometer is designed larger, the detection sensitivity or resolution is lower.

According to the above-mentioned analysis, for different parameters of the structure and levitation control system, performances of the electrostatically suspended microaccelerometer may vary depending on different applications. For instance, the ESMA may be used as following [22]: for microgravity measurements devices, where a range of operation greater than 0.1 g, a resolution of less than 1 μg in a frequency range of zero frequency to 1 Hz are desired; for automotive vehicles, whose stability control system require operation range of ±2 G, resolution <10 mg at DC∼400 HZ frequency range; for inertial navigation, the operation range of ±1 G and resolution <4 μg at DC∼100 HZ range are needed.

#### 2.2.4. Structural Parameters Design of the ESMA Based on Hybrid Microfabrication Method

According to the above analysis, the mass and size of the proof-mass, the capacitive gaps, and the surface flatness of the electrodes and the proof-mass are directly linked to the measurement range, sensitivity and accuracy of the ESMA. A relatively large proof mass, with a thickness of several hundred microns, should be fabricated, which will reduce the Brownian displacement noise, consequently, making the device potentially suitable for the development of a high performance accelerometer, so the accurate microfabrication of large area, small gap spacing capacitors is one of the key techniques for this highly sensitive ESMA device.

**Figure 5.**
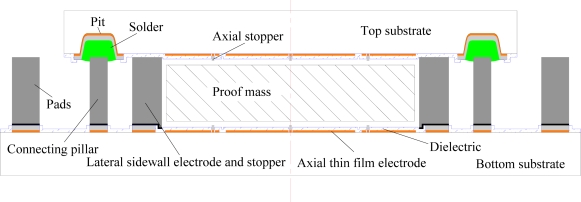
Schematic fabrication structure of the micro-accelerometer.

Microassembly is a critical enabling technology for hybrid manufacturing of complex 3D MEMS devices that utilize non-semiconductor materials and incompatible fabrication processes [23]. It has been widely used to form narrow gaps in microactuators [24]. Based on the microassembly technology, the designed micro-accelerometer can be fabricated using hybrid manufacturing technology: the proof mass and the two stators, *i.e.*, the top stator and bottom stator, are fabricated separately using various machining methods, then they are assembled and solder bonded to form axial and lateral gaps, as the structure shown in Figure 5. The independent thick proof mass can be made of metal such as nickel fabricated using LIGA-like techniques, or monocrystalline silicon fabricated using DRIE, and it can also be made of titanium alloy, Pt-Rh alloy and quartz using precision machining and grinding [1]. For the stators, the hybrid microfabrication techniques can include surface micromachining fabrication of thin film electrodes and interconnections, integration fabrication of thick stator structures using LIGA-like or DRIE micromachining technologies.

As shown in Figure 5, if the height and distance of opposite sidewalls of the lateral electrodes are designed larger than the thickness and side length of the proof mass, respectively, the axial and lateral gap spacing can be determined by their differences. On the other hand, to simultaneously increase displacement sensing resolution and electrostatic loading capacity, the electrode design there must be a trade off between levitation electrodes and common electrodes. That is, according to the detection current *i_p_* expression (40) and the maximum measurable acceleration expression (46), in the first place, the nominal capacitance *C_z0_* and *C_s0_* must be large enough for sensing and levitation, mainly to meet maximum acceleration with a specified actuation voltage. Then the common capacitance *C_c0_* should be designed large enough to maximize the coefficient *K_c_*. A multi-objective optimization design model of the structural parameters of the ESMA can be established as [25]. Here the area of common electrode is optimally designed as about 47 percent of the total axial thin film electrodes area. The main design parameters of the ESMA, based on LIGA-like and DRIE micromachining technologies, are summarized in Table 1.

## 3. Fabrication Processes of the Designed ESMA

In this paper, the main structural parameters illustrated in Table 1 were employed to fabricate the first prototype of the 6-DoF micro-accelerometer, which is mainly using for on-ground experiment. For the microfabrication of this prototype, a silicon proof mass of 450 μm thickness made using the DRIE technique and the integrated thick nickel structures of the bottom stator made using the SU-8 UV-LIGA technique were adopted. The formed nominal gaps for axial and lateral directions were designed of 4 μm and 10 μm, respectively.

As the structure shows in Figure 5, the microfabrication processes of the two stators, the proof mass and the bonding were described below. For the top stator wafer process, firstly, on the Pyrex substrate, positioning pits, required for later bonding the two stators by soldering connecting pillars, are wet etched. Then, using surface micromachining process, thin film electrodes and interconnections, nickel stoppers, solder in positioning pits, and alumina passivation film are fabricated through photolithography, electroplating, sputtering and etching. Like the top glass wafer, on the bottom glass wafer, the thin film electrodes and interconnections, nickel stoppers and foundations of later electroformed thick structures [26], and alumina dielectric passivation film are fabricated by surface micromachining processes. Then, the alumina passivation film is patterned to expose the foundations and thick nickel structures, such as lateral electrodes, connecting pillars and pads, are electroformed through the patterned SU-8 thick resist mold. After a lapping step, the thick resist mold and seed layer are removed, which ends the process of fabrication of the bottom stator. A monocrystalline silicon proof mass is fabricated by DRIE using ICP etching machine and coated with Cr/Au conductive layer by sputtering deposition. The fabricated bottom stator, with the proof mass embedded by assembly, is aligned with the top stator; then they were bonded by reflow of the solders. So, the axial and lateral gaps are formed and the micro-accelerometer chip is obtained.

In the following, the microfabrication processes of the two stators, the proof mass and the bonding are described, respectively, and the key fabrication steps are explained in detail.

### 3.1. Microfabrication Process of the Top Stator

**Figure 6.**
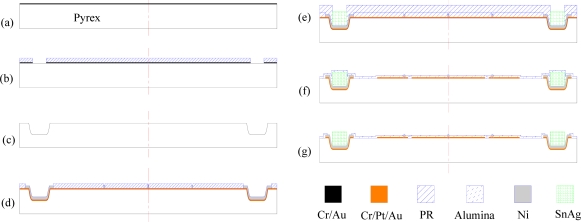
Process flow of the top stator.

To fabricate the top stator of the ESMA, special attention should be paid to the wet etching of the positioning pits, because by metallization of the pits, such as in electroplating, the top stator can be solder joined with the pillars of the bottom stator, which provides signal routes to the pads of the bottom stator. For metallization, the deep etched pits should have sloped walls with smooth surfaces and a small ratio of lateral undercutting to the etch depth. Borosilicate glass, such as Pyrex, can be wet etched with better pits or grooves profile, so a Pyrex 7740 wafer is used as the top substrate in this work. The fabrication flow of the top Pyrex wafer is simply illustrated in Figure 6, which can be divided into two parts.

The first part, as shown in Figure 6(a–c), is mainly to etch the glass wafer to form positioning pits, into which pads and solder will be formed in the later part. When deep wet etching of Pyrex with HF-based etching solution, defects such as edge notches, pinholes and rapid lateral underetching, often happen [27]. The main reason is due to the defects, poor adhesion, and residual stress in the mask layer. In this paper, to get wet etched pits of a depth of near 30 μm, with smaller ratio of underetching to etch depth, the sputtering deposited Cr/Au with thickness of 40 nm/300 nm was adopted.

As shown in Figure 6(a,b), window patterns of the photomask were transferred onto the Pyrex substrate by means of a combination of photolithography, metal film mask deposition and etch steps. Firstly, the wafers were cleaned in H_2_O_2_-H_2_SO_4_ (1:2 vol. parts), rinsed with deionized water and then dried. Secondly, as sputtered thin films were reported to have better performance as etch masks due to their high film density and better adhesion, in this work, magnetron sputtering was employed to deposit a 40 nm thick Cr adhesive layer and a 300 nm gold layer as metal etch mask [Figure 6(a)]. Then, a 10 μm thick photoresist layer (AZ4620, Clariant) was photolithographically patterned, followed by ion beam etching of the Cr/Au mask. Hereto the combination Cr/Au/PR (40 nm/300 nm/10 μm) wet etching mask was obtained, as illustrated in Figure 6(b). A mixture solution of 49% hydrofluoric acid-70% nitric acid:water (20:14:66 vol. parts) was used to etch the Pyrex [27]. In a water bath at 40 °C, the Pyrex was etched for 30 min with a slow agitation, which resulted in near 30 μm deep pits. Then the combination mask was removed [Figure 6(c)].

The second part is a surface micromachining process, as shown in Figure 6(d–g), where axial stoppers, solder, thin film electrodes and interconnections, and dielectric layers were formed. Firstly, a Cr/Pt/Au (20/80/100 nm) seed layer was deposited on the Pyrex substrate by sputtering deposition. Then, the first photoresist layer was photolithographically patterned, and using this Cr/Pt/Au film as plating base, nickel axial stoppers and under-bump metal (UBM) in the etched pits, in thickness of 2 μm, were electroplated, as shown in Figure 6(d). Following the patterning of the second photoresist layer, lead-free tin-silver (Sn/3.0Ag) solder was electroplated from the nickel UBM into the pits by using Slotoloy SNA 30 electrolyte (Schloetter Plating Inc.) [Figure 6(e)]. After that, using the patterned photoresist as a mask, the Cr/Pt/Au film was then etched by ion beam etching to form axial electrodes patterns. A 0.5 μm alumina passivation layer was then deposited over the structure using a sputtering machine [Figure 6(f)]. Finally, to expose the stoppers and the solder, the alumina layer was patterned with a 65 °C phosphoric acid [Figure 6(g)], thus completing the fabrication process for the top stator.

### 3.2. Microfabrication Process of the Bottom Stator

For bottom stator fabrication of the gyroscope, the surface micromachining process was employed to form stoppers, thin film electrodes and the interconnections. Then, using SU-8 ultrathick photoresist as electroplating mold, the thick nickel structures, such as pillars, lateral electrodes and stoppers, were integrated with the fabricated circuitry on the same chip. The key fabrication technique of the bottom stator was the integration of thick nickel structures with the bottom structures by successful removal of SU-8 mold.

SU-8 has been used extensively in making high aspect ratio MEMS device structures [13]. A principle application of SU-8 is utilized as electroplating molds for high aspect ratio microstructures in x-ray LIGA or UV-LIGA process. Unfortunately, when used as a mold material, the highly cross-linked SU-8 is difficult to remove or strip by conventional means [28]. This hard removal of SU-8 mold has dramatically limited its wide application in making high aspect ratio microstructures, especially in making integrated microstructures with surface microfabricated components. Using the fuming sulfuric acid oxidizing method for removal of SU-8 mold and metal foundations constructed to consolidate the electroplated structures, we have successfully integrated more than 200 μm thick nickel structures [9]. The fuming sulfuric acid can etch organic material by oxidizing and dehydration while causing minimal corrosion to most metals due to surface passivation, which makes it an inexpensive and effective way to remove SU-8 molds with integrated metal structures. In this paper, thick nickel lateral electrodes, connecting pillars and pads of the ESMA, in thickness of about 500 μm, were integrated with the surface micromachined structures using this SU-8 removing technique.

**Figure 7.**
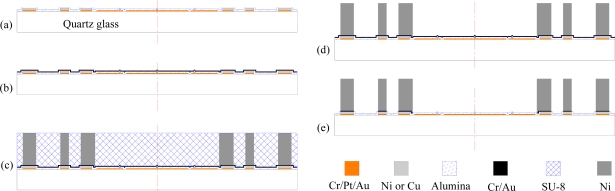
Schematic diagram of the fabrication process of the bottom stator.

A schematic diagram of the fabrication process of the bottom stator, using quartz glass as substrate, is shown in Figure 7. Like the fabrication process of the top glass wafer shown in Figure 6(d–g), on the bottom glass wafer, the Cr/Pt/Au thin film electrodes and interconnections, nickel or copper stoppers and foundations, and alumina passivation film were fabricated by a surface micromachining process through photolithography, electroplating, sputtering and etching [Figure 7(a)].

For subsequent integration of thick nickel microstructures, a 0.5 μm alumina passivation layer was patterned with phosphoric acid to expose the surface of the metal foundations, on which thick Ni structures would be electroformed. To obtain good step coverage, a 30/200 nm Cr/Au layer, serving as a seed layer for electroplating, were deposited via sputtering [Figure 7(b)]. Then the thick SU-8 resist was spin-coated and patterned defining molds for Ni structures, namely pads, pillars, lateral electrodes and stoppers. Table 2 summarizes the process parameters and the related equipment used in generating about 500 μm thick SU-8 molds.

On the exposed areas of the Cr/Au seed layer, the Ni structures were electroplated in a nickel-sulfamate based solution. The electroformed Ni structures, together with the SU-8 mold, were planarized by a grinding step [Figure 7(c)]. In the SU-8 mold removal process, the lapped wafer was immersed in fuming sulfuric acid (H_2_SO_4_·xSO_3_), with 40% free sulfur trioxide content, holding in airtight glass container. During etching and oxidizing process, the cross-linked SU-8 mold was broken into gel-like tiny pieces. With the penetration of the acid, the SU-8 mold was gradually etched from the surface to the depth. For complete etching removal of the about 500 μm thick SU-8 molds, the wafer was continuously immersed for about 4 hours. Finally, the etched residue was cleaned in acetone and DI water with ultrasonic vibration. After removing the SU-8 mold, the lapped Ni structures standing on the substrate were released [Figure 7(d)]. To isolate the Ni structures, the Cr/Au seed layer was removed by plasma sputtering [Figure 7(e)].

### 3.3. Microfabrication of the Gold Coated Silicon Proof Mass

The proof mass fabrication process was relatively simple because only one lithography step was needed. However, a flat proof mass with vertical sidewalls and smooth surfaces had to be carefully fabricated. In this paper, a monocrystalline silicon proof mass was micromachined by DRIE using an Alcatel 601E machine. Firstly, a double-side polished, n-type Si (100) wafer, in a designed thickness of the proof mass, was bonded with an accompanying wafer, using 4 μm benzocyclobutene (BCB, Dow Chemical) void-free coating as the adhesive material [29]. With Kral Suss SB6 bonder, the two wafers, one of which was coated with the BCB, were joined by curing the BCB with applied pressure in vacuum environment. Secondly, a 20 μm photoresist was patterned as an etching mask, followed by deep reactive ion etching through the silicon wafer to the BCB layer using the Bosch process. Then, a piranha solution (3:1 mixture of concentrated sulfuric acid with hydrogen peroxide), was used to remove the cured BCB and release the silicon proof masses. Lastly, the proof mass was wholly coated with Cr/Au conductive layer by double-sided sputtering deposition.

### 3.4. Assembly and Reflow Bonding

For the assembly, each released proof mass, with required sizes of side length and thickness, was put into each bottom stator which has been thinned by grinding and polishing with controlled height of the lateral electrodes; then, the bottom stator was aligned with the top stator through the transparent glass substrate. They were then clamped by a jig, which would provide a pre-tightening force for tight contacting between the top stator and top surface of the lateral electrodes after reflow bonding, consequently, the designed axial gaps would be ensured. Finally, the two clamped stators were bonded by reflow of SnAg solders at N_2_ atmosphere in a reflow oven, with a peak temperature of about 260 °C. This also provided an automatic self-alignment of the two stators due to the surface tension during the reflowing process. This bonding could be wafer-level or chip-level.

## 4. Fabrication Results of the ESMA

**Figure 8.**
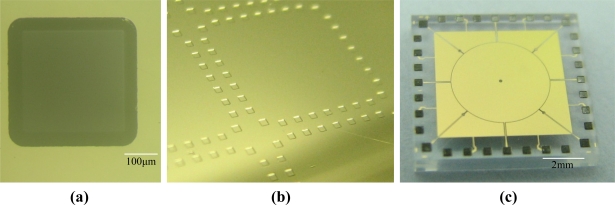
Fabrication results of the top stator: **(a)** Microphotograph of one of the etched pits after removal of photoresist mask; **(b)** Photograph of partial wafer after Cr/Pt/Au layer deposited; **(c)** Photograph of one diced top stator, with SnAg electroplated within the pits.

A microphotograph of one of the etched pits after removal of the photoresist is shown in Figure 8(a). A photograph of a partial wafer after the Cr/Pt/Au seed layer deposited is shown in Figure 8(b). It can be found that no visible defects were formed and the etched pits had smooth surfaces with sloped sidewalls. The pit, with photomask window of 300 × 300 μm^2^, was measured to be about 27 μm in depth and laterally, the undercutting was only 38 μm. In these etched pits, the nickel pads and SnAg solder were successfully electroplated. An optical photograph of one die of the fabricated top stator is seen in Figure 8(c).

**Figure 9.**
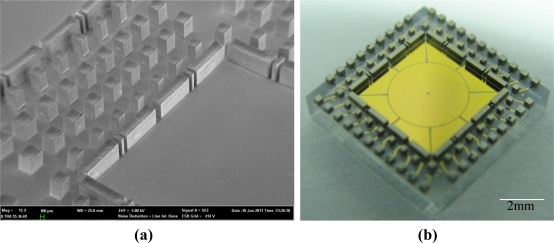
Fabrication results of the bottom stator: **(a)** SEM image of the integrated nickel structures, in thickness of about 500 μm, after removal of the SU-8 mold; **(b)** One die of the fabricated bottom stator, whose size is 10 × 10 mm^2^.

For the fabrication of the bottom stator, as the SEM image in Figure 9(a) shows, plated Ni structures with a thickness of about 500 μm were successfully released and remained on the wafer after removing the SU-8 mold. It can be seen that no residues remained on the high aspect ratio nickel structures. An optical photograph of one die of the fabricated bottom stators, with ultrathick lapped nickel structures integrated, is seen in Figure 9(b). Therefore, the fuming sulfuric acid oxidizing method is an effective way to remove ultrathick SU-8 electroplating molds for integrating 3D thick microstructures with surface micromachined structures.

**Figure 10.**
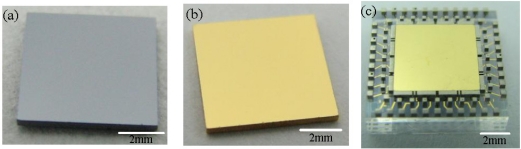
**(a)** Optical photograph of one silicon proof mass released made through DRIE. **(b)** Silicon proof mass coated with Cr/Au layer, size: 6 mm × 6 mm × 450 μm; **(c)** photograph of a diced bottom stator with a proof mass embedded.

Optical photographs of one of the silicon proof masses, made from the double-side polished wafer through DRIE then coated with sputtering deposited Cr/Au conductive layer, are shown in Figure 10(a,b), respectively. The proof mass, with a size of about 6 mm × 6 mm × 450 μm, shows flat and smooth surfaces. Figure 10(c) shows a photograph of a diced bottom stator with a proof mass embedded, where the axial gap spacing of the ESMA was defined by the distance between top surfaces of the grinded sidewall electrodes and the proof mass. For example, the released 500 μm thick nickel structures were carefully ground and polished to a height of 458 μm, so a 4 μm nominal axial capacitive gap was formed in the ESMA.

**Figure 11.**
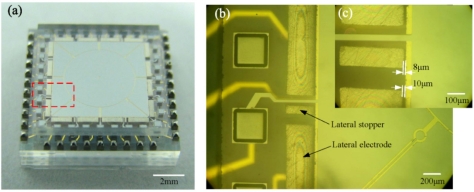
**(a)** Optical photograph of a solder bonded microaccelerometer chip about 10 × 10 × 3 mm^3^, where the pads are coated with solder bumps. **(b)** and **(c)** Microphotographs of the partly enlarged microaccelerometer chip, seen through the bonded top glass stator.

After the three parts, namely top stator, bottom stator and the proof mass, were assembled and the two stators were bonded by solder reflow, an ESMA chip about 10 × 10 × 3 mm^3^ in size, forming lateral gap of 10 μm and axial gap of 4 μm, was obtained, as the optical image in Figure 11(a) shows. Its partly enlarged microphotographs are shown in Figure 11(b,c). The formed lateral gaps were measured by optical microscope, and the gap between the lateral stopper and the proof mass was 8 μm. As partly shown in Figure 11(b,c), the uniform axial gap was ensured by observation of interference fringes around the contact area between each lateral electrode and the top glass stator. The height of axial stoppers was measured about 1.2 μm as determined using a profile meter. The results show that by employing hybrid microfabrication techniques, the prototype of the designed microaccelerometer was successfully realized.

## 5. Levitation Experiments of the ESMA

### 5.1. Levitation Experiment System

**Figure 12.**
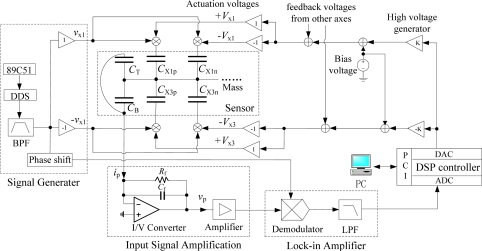
Schematic diagram of detection and control system for the ESMA, taking differential electrode pairs X1(X_1p_, X_1n_) and X3(X_3p_, X_3n_) for example.

**Figure 13.**
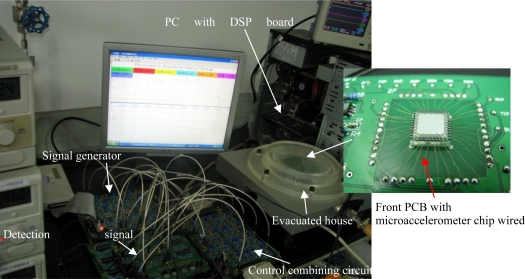
Levitation experiment system for the fabricated micro-accelerometer.

A schematic diagram of close-loop detection and control system for the ESMA sensor, which mainly consists of micro-displacement detection unit, DSP control unit and voltages combining unit, is illustrated in Figure 12. The micro-displacement detection unit is composed of a multi-frequency signal generator, input signal front-end amplification and lock-in amplifier [15]. The voltages combining unit includes formation of actuation voltages and their superposition of carrier voltages. Correspondingly, the levitation experiment equipment for the fabricated ESMA is shown in Figure 13. It is mainly composed of four PCBs, a DSP board and a PC upper machine. The four PCBs are used for signal generator, front-end amplification, displacement signal processing and voltages combining circuits, respectively. The front-end amplification PCB with the ESMA chip wired was housed in an evacuated chamber, whose vacuum created by mechanical pump is about 2,000 Pa.

Employing an AD9832 as direct digital synthesis (DDS) chip controlled by the 89C51 microcontroller, together with band pass filter (BPF) and phase shifter circuits, the signal generator can produce multi-frequency sine waves with changeable phases. These signals, with frequencies in the range of 100∼220 KHz, are used as 6-axis displacement detection carriers and reference waves for modulation and demodulation, respectively. By applying sensing signals to the control electrodes, the small AC current detecting signal *i_p_*, which indicates the position of the proof mass along the multiple axes, is obtained from the common electrode via the proof mass and changed to AC voltage signal *v_p_* through a low-noise charge amplifier (AD8610). It is then amplified by a low-noise wideband amplifier (AD811) and applied to the lock-in amplifier, where phase-sensitive demodulation (AD734) is performed to obtain the maximum output and then low-pass filtered with MAX274 to achieve DC voltage detecting signals. Based on these DC signals, a 32-bit floating-point DSP processor (TMS320VC33), with a sampling rate of 10 kHz, is used as a digital controller to produce position control voltages employing PID control algorithm. In the same DSP board, there are 14-bit analog-to-digital converters with an input range of ±5 V, 12-bit digital-to-analog converters with ±10 V output voltage range, and 32-bit PCI interface circuits for communication with PC machine. By amplifying the control voltages, the outputs of high voltage generators (OPA445) are summed with a DC bias voltage and feedback voltages from the other axes, consequently, the actuation voltages are generated. Then they are applied to the corresponding differential electrode pairs to keep the proof mass in the null position. Taking the lateral differential electrode pairs X1(X_1p_, X_1n_) and X3(X_3p_, X_3n_), shown in Figure 12, for instance, the actuation voltages ±*V_x1_*, ±*V_x3_*, expressed as (19), are composed of bias voltage *V_x0_*, feedback voltages of *U_x_* and *U_ϕ_*. They are simultaneously superimposed with carrier signals ±*v_x1_* to make close-loop position control of the proof mass caused by displacement *x* and rotation *ϕ*.

### 5.2. Results of Levitation Experiment

**Figure 14.**
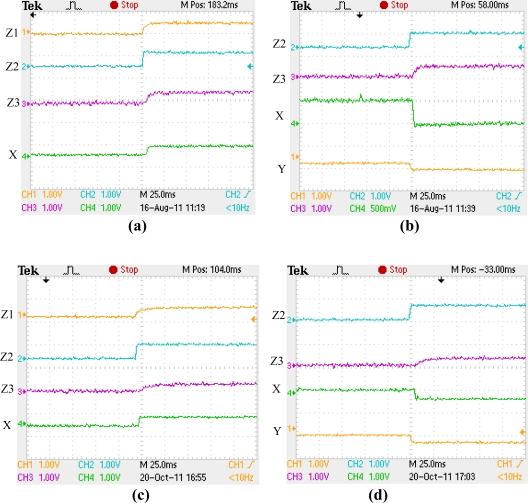
Electrostatic levitation displacement curves of the proof mass: **(a)** Curves for Z1, Z2, Z3 and X positions; **(b)** Curves for Z2, Z3, X and Y positions; **(c)** and **(d)** repetitions of levitation displacement curves of (a) and (b) after about two months.

At present, stable full levitation control of the proof mass has been demonstrated, as levitation displacement curves shown in Figure 14.

Since the Tektronix oscilloscope employed has only four channels, position response curves of the proof mass, in form of detection voltages, for channel Z1, Z2, Z3 and X positions are illustrated in Figure 14(a), while for channel Z2, Z3, X and Y positions are shown in Figure 14(b), which were obtained from two levitation-process experiments, respectively.

Here the proof mass was levitated against the force of gravity along the Z-axis direction from its seated axial stoppers to some given suspension position. Z1, Z2 and Z3 were the three differential capacitance positions of four differential capacitances formed between Z-axis levitation electrodes and the proof mass, as shown in Figure 1. The position responses of the proof mass along X axis and Y axis were controlled to suspend it at some position during the Z-axis levitation process. The applied bias voltages during the levitation process were 35 V for Z-axis direction and 40 V for X-axis or Y-axis direction. For the two levitation processes as shown in the Figure 14(a,b), respectively, the suspension targets of Z2 and Z3 positions were the same while motion directions for X axial target positions were opposite. It is found that, for each direction or position of the proof mass, fast initial levitation process within 10 ms, and stable suspension at the given target position, were achieved. From the levitation curves of the Z2 and Z3 positions, we can see that repeatability of the levitation process was very good and the influence of cross-axis motions, such as influence on Z-axial motion caused by X- or Y-axial motion, was very small. From suspension stability test of the levitated proof mass for over eight hours, we found there were no visible deviation from the target suspension position happened. After more than two months passed, we repeated the levitation processes of Figure 14(a,b), and its levitation displacement curves are shown in Figure 14(c,d), respectively. The results shows that the levitation process to the same given target position was well repeated.

## 6. Conclusions

In this paper, a designed structure of a micromachined electrostatically suspended six-axis microaccelerometer, with a square plate as proof mass housed by top stator and bottom stator, was presented. Its detailed operation parameters such as the capacitances and electrostatic forces/moments, detection and levitation control of the proof mass position, acceleration measurement and main performance indexes, structural parameters design using hybrid microfabrication method, were presented. A precision ESMA prototype chip was achieved using hybrid microfabrication techniques, that is, the top stator and the bottom stator were fabricated by surface micromachining and UV-LIGA integration fabrication of thick nickel structures using SU-8 photoresist, the gold coated silicon proof mass of 450 μm in thickness was mainly obtained by DRIE, and then they were assembled and solder-reflow bonded. At the bottom stator, by successful removal of SU-8 mold using fuming sulfuric acid oxidizing method, the nickel structures about 500 μm thick were integrated with the surface micromachined structures such as axial stoppers, electrodes and interconnections. Levitation control experiments show that fast initial levitation within 10 ms and stable full suspension of the proof mass for the fabricated ESMA chip were successfully demonstrated. At present, linear and angular acceleration measurement and calibration of this microaccelerometer are ongoing. For higher microfabrication precision and smaller lateral gap spacing formation, the x-ray LIGA technique will be employed in further research. The hybrid microfabrication techniques described in this paper can be applied for fabrication of other complicated 3D MEMS devices.

## Figures and Tables

**Table 1.** Main design parameters of the first prototype of ESMA.

**Parameters**	**Design values**
Basic side length of the proof mass, *l*/mm	6
Basic thickness of the proof mass, *h*/μm	450
Mass of the proof mass, *m*/mg	43.74 (silicon), 144.18 (nickel)
Main moment of inertia, *I_z_*/10^−10^ kg m^2^	2.624 (silicon), 8.651 (nickel)
Moment of inertia around x- or y-axis, *I_x_*/10^−10^ kg m^2^	1.320 (silicon), 4.350 (nickel)

Nominal axial gap, dz0/μm	3∼5 (vary with the lapped height of the lateral elctrodes)

Nominal lateral gap, ds0/μm	3∼12 (vary with small changes of side length of the proof mass)

Area of each axial levitation electrode Az/mm^2^	2.288

Area of each common electrode, AC/mm^2^	16.183

Area each lateral levitation electrode, As/mm^2^	0.586

Capacitance of all common electrodes,CC0/pF	71.639(dz0 = 4 μm)

Capacitance of one axial electrode pair, Cz0/pF	10.129(dz0 = 4 μm)

Capacitance of one lateral electrode pair, Cs0/pF	1.020(ds0 = 10 μm for UV-LIGA)

	2.550(ds0 = 4 μm for X-LIGA)

Bias voltage for axial suspension, Vz0/V	10 ∼ 40

Bias voltage for lateral suspension, Vs0/V	20 ∼ 50

**Table 2.** Process conditions of SU-8 2100 (from MicroChem) with mold height of about 500 μm.

**Process step**	**Parameters**	**Equipment**
Spin-coating	Spread: 200 rpm ,15 s	Karl Suss RC8
	Spin: 700 rpm, 20 s	
Soft bake	65 °C, 30 min; 95 °C, 120 min	Hotplate
Exposure	Dose 2.6 mW/cm^2^, 250 s	Karl Suss MA6
Post-expose bake	65 °C, 30 min; 90 °C, 40 min	convection oven
Development	40 min immersion in PGMEA	
RIE cleaning	O_2_ 35 sccm, pressure 5 Pa, 25 W, 300 s	Alcatel Nextral 100
